# Functional Morphology of Leg Mechanosensory Organs in Early Postembryonic Development in the Stick Insect (*Sipyloidea chlorotica*)

**DOI:** 10.3390/insects15060392

**Published:** 2024-05-28

**Authors:** Johannes Strauß

**Affiliations:** 1 AG Integrative Sensory Physiology, Institute for Animal Physiology, Justus Liebig University Gießen, 35392 Gießen, Germany; orthopteroid.neuro@gmail.com; Tel.: +49-641-99-35253; 2 Center for Mind, Brain and Behavior (CMBB), University of Marburg and Justus Liebig University Gießen, Hans-Meerwein-Straße 6, 35032 Marburg, Germany

**Keywords:** chordotonal organ, mechanoreceptor, biomechanics, functional morphology, vibration receptor, subgenual organ, ontogeny

## Abstract

**Simple Summary:**

Stick insects and their mechanoreceptors are important models for neurobiology and physiology. Mechanoreceptors are sensory systems that detect mechanical stimuli like wind, airborne sound, substrate vibration, or gravity. They are present in insects in highly diverse forms. Here, the morphology of a mechanosensory complex (the subgenual organ complex) with two distinct organs in the legs of a stick insect (*Sipyloidea chlorotica*) is described for animals that were newly hatched. The overall organisation of this sensory complex is unique in stick insects, and the present study extends the current knowledge from adult insects to the early juvenile instars. The morphological results show a connection between the two sensory organs (subgenual organ and distal organ) by a thin membrane, which had already been documented in adult stick insects. By the time of hatching, both the sensory neurons activated by mechanical stimuli as well as further tissues in the sensory complex are present, and they likely develop during embryogenesis. The sensory organs could also be functional at hatching. This report discusses the implications of a connection between the subgenual organ and the distal organ, suggesting that the membrane would influence the displacements of the two organs.

**Abstract:**

The subgenual organ complex of stick insects has a unique neuroanatomical organisation with two elaborate chordotonal organs, the subgenual organ and the distal organ. These organs are present in all leg pairs and are already developed in newly hatched stick insects. The present study analyses for the first time the morphology of sensory organs in the subgenual organ complex for a membrane connecting the two sensory organs in newly hatched insects (*Sipyloidea chlorotica* (Audinet-Serville 1838)). The stick insect legs were analysed following hatching by axonal tracing and light microscopy. The subgenual organ complex in first juvenile instars shows the sensory organs and a thin membrane connecting the sensory organs resembling the morphology of adult animals. Rarely was this membrane not detected, where it is assumed as not developed during embryogenesis. The connection appears to influence the shape of the subgenual organ, with one end extending towards the distal organ as under tension. These findings are discussed for the following functional implications: (1) the physiological responses of the subgenual organ complex to mechanical stimuli after hatching, (2) the influence of the membrane on the displacement of the sensory organs, and (3) the connection between the subgenual organ and distal organ as a possible functional coupling.

## 1. Introduction

Chordotonal organs are mechanosensory organs of insects and crustaceans [1,2]. These organs are present in all parts of the insect body [1,3,4]. Chordotonal organs consist of a highly variable number of scolopidial sensilla that can range from a single sensillum to several thousand sensilla [5,6,7,8,9,10,11,12,13]. Scolopidial sensilla detect mechanical forces acting on the dendrite of the sensory neurons [3,14,15]. This mechanical stimulation is mediated by the structure of the sensory neurons and additional cell types associated with the neurons. These cells are, in particular, the attachment cell and the scolopale cell producing the scolopale surrounding the neuron’s dendrite [14,15,16,17]. The sensory organs can be connected to diverse parts of the body, such as cuticle elements, tendons, or trachea. Depending on the connection to other structures, different mechanical stimuli can activate the sensory organs: the mechanical coupling determines how mechanical stimuli reach the sensilla and thus how the sensory organ is activated [18]. Resulting from the different connections, chordotonal organs can detect external stimuli such as substrate vibrations and sound or stimuli generated by body movements such as stretching or bending [1,17,19,20]. 

**Figure 1:**
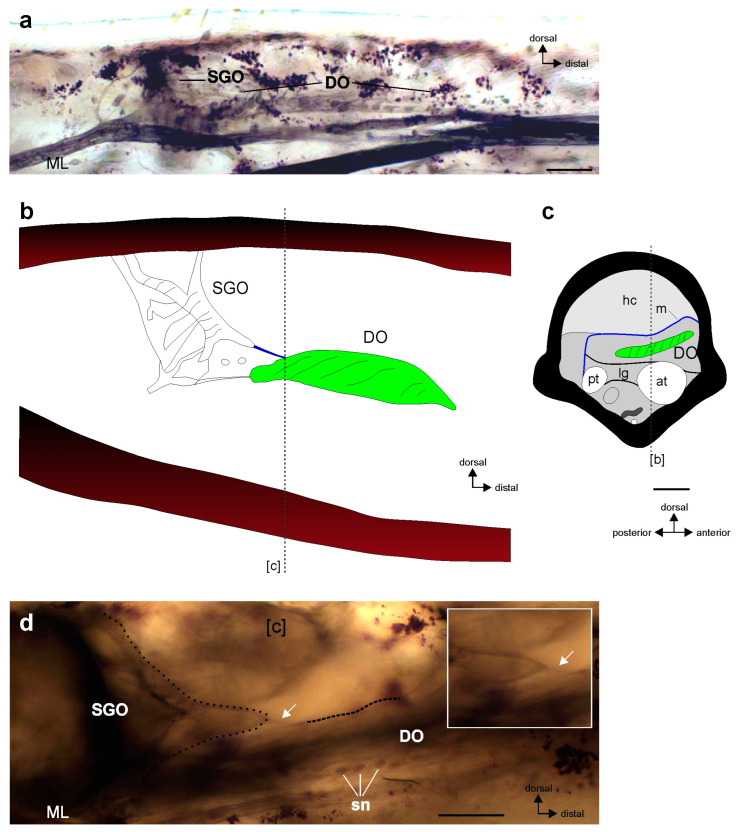
The subgenual organ complex in adult *Sipyloidea chlorotica*. (**a**) Neuroanatomy of the subgenual organ complex (midleg); viewed from anterior. Sensory neurons and nerve branches have been stained. The complex consists of the subgenual organ (SGO) and distal organ (DO); note the different orientations of these sensory organs. (**b**,**c**) Schematic of the subgenual organ complex of the midleg, redrawn from microcomputed tomography sections [38]. The DO is shown in green, the membrane in blue. The different levels of sections are indicated by the hatched lines. (**b**) The SGO and the DO are arranged almost perpendicularly to each other and are connected by the membrane (m) (shown in blue), in vertical longitudinal section. Other internal tissues are omitted. [c] indicates the section level shown in Figure 1c. (**c**) Schematic of the tibia at the level of the proximal DO, in transversal section. The connecting membrane is located in the central tibia, dorsally to the DO. [b] indicates the section level shown in Figure 1b. (**d**) The connecting membrane (white arrow) between the SGO (outlined by the dotted line) and the DO (dorsal part outlined by the hatched line) (midleg), viewed under light microscopy, shown in anterior view. Note the shape of the SGO (inset) extended in distal direction of the tibia. Scale bars: (**a**,**d**) = 100 µm. Abbreviations: at, anterior trachea; DO, distal organ; hc, haemolymph channel; lg, ligament; m, membrane; ML, midleg; pt, posterior trachea; SGO, subgenual organ; sn, cell bodies of sensory neurons.

The tibia of Polyneoptera houses the subgenual organ complex, which contains the subgenual organ that detects substrate vibrations and also airborne sound, and a few associated chordotonal organs [1,21]. These sensory organs usually show morphological differences in their position and attachments [22,23,24,25]. The stimulus transfer to the sensilla depends on the connection of sensory organs to other structures like the leg cuticle or the position in the haemolymph channel [22,26,27]. The complexity of sensory structures increases by connections between individual sensilla, sensilla groups, or sensory organs. The morphological connection between sensory organs is also functionally relevant with respect to physiological similarities, e.g., in frequency tuning to vibration stimuli [27]. Such a morphological connection is documented in the subgenual organ complex of stick insects (Phasmatodea), which consists of two chordotonal organs, the subgenual organ (SGO) and the distal organ (DO) located in close proximity to each other [21,28]. These chordotonal organs are placed in the haemolymph channel, and it is likely that both are activated by substrate vibrations transmitted through the haemolymph [21]. The organs are connected by a thin membrane between the SGO and the DO, which is present in all leg pairs [21,29,30]. This membrane is located between the distal SGO and the DO, and the morphology of the subgenual organ complex in adults is shown in an overview in Figure 1. The membrane between the sensory organs merges with the DO on its dorsal surface (Figure 1b,d). Neither the ultrastructural or the biochemical properties nor the functional relevance of this membrane have so far been investigated. However, the membrane may have a mechanical influence on both sensory organs. The morphological findings have also shown that the SGO is stretched in the distal direction, resulting in a characteristic extension of the SGO towards the DO (Figure 1b,d). This morphology suggests that the membrane may provide a relevant coupling between the SGO and the DO [29]. It does not contain neuronal elements, shown by staining of neuronal structures through axonal tracing [29,30]. 

So far, the membrane has been analysed in adult stick insects of only a few species. As hemimetabolous insects, Phasmatodea undergo a number of postembryonic moults between successive juvenile instars [31,32]. Mechanical stimuli including substrate vibrations detected by the SGO are relevant to mediating mating, territoriality, or predator detection in insects [33,34]. From the time of hatching, substrate vibrations can be transferred to leg mechanoreceptors, and such stimuli originating, for example, from predators would be important to detect not only for adult insects but also for juvenile instars. Obviously, the morphological structures relevant to the mechanosensory function of the sensory complex also include cells and tissues such as the accessory cells or attachment structures [1,15,35]. In stick insects, the chordotonal organs differentiate during embryonic development (*Carausius morosus* (Brunner von Wattenwyl 1907) [36]). The sensilla in the subgenual organ complex in orthopteroid insects are also physiologically functional in early juvenile instars since they respond to vibrational stimuli (as shown for bush crickets [Tettigoniidae] [37]). Therefore, the subgenual organ complex in juveniles would require not only sensory neurons but also connecting structures, including the membrane between sensory organs.

In *Sipyloidea chlorotica* (Audinet-Serville 1838), syn. *S. sipylus* (Westwood 1859) [39], the sensory organs were investigated here in the first juvenile instar. A neuroanatomical analysis previously showed that the sensory organs are differentiated in newly hatched instars [40]. So far, the additional structures such as the membrane between the sensory organs have not been documented. In this study, it is hypothesized that not only the sensory neurons but also the tissue of the SGO/DO and the membrane connection between the sensory organs are differentiated during embryogenesis and are thus present after hatching. Therefore, the presence of the membrane connection between the sensory organs is analysed in this study in the first postembryonic stage in *S. chlorotica*, and the morphology is predicted to be similar to that of adult insects [21]. Among stick insects, *S. chlorotica* is an important laboratory species [32]. It was previously studied for the neuroanatomy of the subgenual organ complex. The very light cuticle lacking prominent pigmentation allows for neuroanatomical analysis [28,40,41,42], and the membrane in the subgenual organ complex was described in adult individuals [21]. If the connecting membrane is present in newly hatched juveniles, this would also support the physiological function of mechanoreceptors in the subgenual organ complex in the postembryonic stages. 

## 2. Materials and Methods

### 2.1. Insects

Female *Sipyloidea chlorotica* (Audinet-Serville 1838) were taken from a colony of parthenogenically reproducing stick insects at the Institute for Animal Physiology, Justus Liebig University Gießen. The colony was reared at a room temperature ranging between 21 and 23 °C. The insects were provided with leaves of bramble ad libitum as food, and water was provided daily by spraying. 

**Figure 2:**
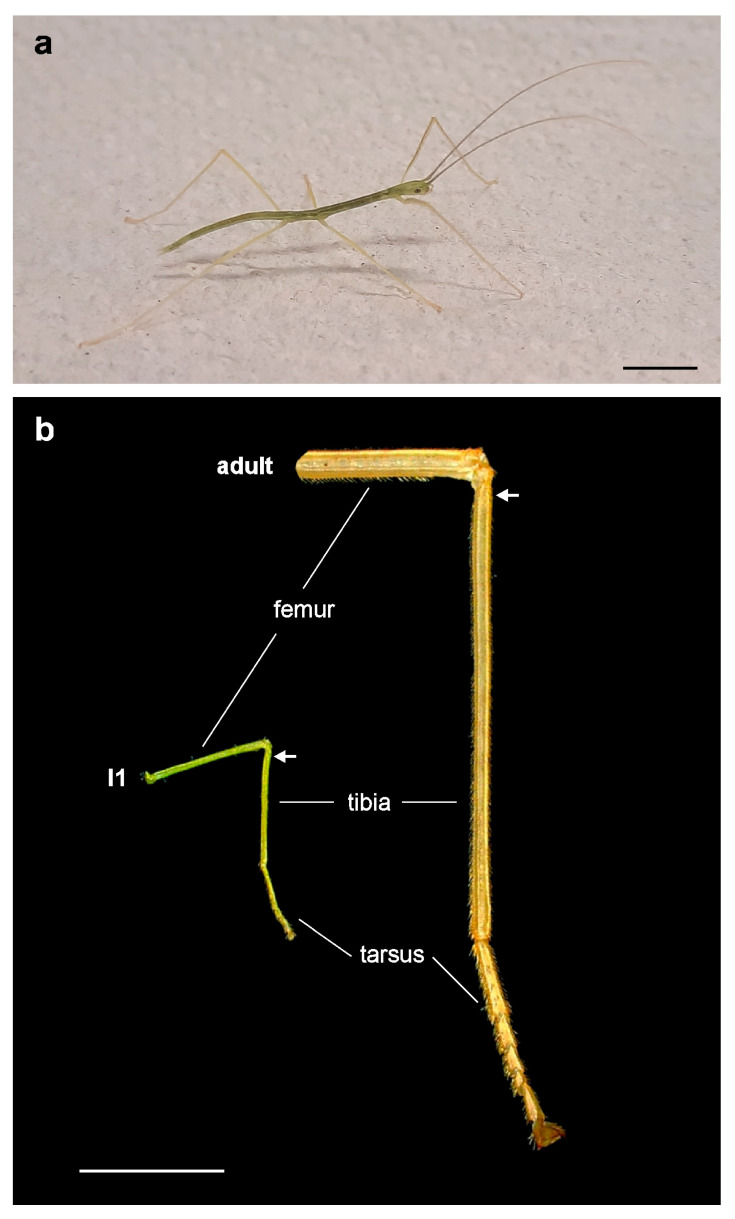
Habitus of *Sipyloidea chlorotica*. (**a**) Individual of *S. chlorotica*, first juvenile instar (18 mm body length). (**b**) Comparison of the leg size (midleg) of a first juvenile instar (I1; left) and an adult stick insect (right). White arrows indicate the level of the tibia locating the subgenual organ complex. The legs are shown in anterior view. Scale bars = 0.5 cm.

The adults and juvenile stages were identified based on morphological features and body length, as described by Carlberg (1987) [43]. Newly hatched individuals (Figure 2a) were identified as described previously [40] by keeping eggs in plastic containers and collecting insects for preparation after hatching at night, which is common for stick insects. Preparations using axonal tracing were carried out directly on the morning after hatching. The body length of the first juvenile instars was between 18 and 20 mm, consistent with data from Carlberg (1987) [43]. In the present study, newly hatched stick insects were analysed since a staging system for the embryonic development of *S. chlorotica* is not established. The chordotonal organs in the legs, which differentiate during embryogenesis [36], could most likely detect mechanical stimuli after hatching, when the legs are used in locomotion, touch, and vibration detection. The leg preparations analysed in this study were obtained from adult insects (*n* = 9; midlegs), first juvenile instars using axonal tracing (*n* = 35), and first juvenile instars without axonal tracing (*n* = 15) (see below for details). The leg preparations of first juvenile instars using axonal tracing were previously studied for the neuroanatomy of sensory organs [40] and were stored in methyl salicylate. From the axonal tracing experiments, 35 leg preparations were analysed (7 foreleg preparations, 21 midleg preparations, and 7 hindleg preparations). All leg pairs were included since the organization of the subgenual organ complex is identical in all thoracic legs of first juvenile instars in *S. chlorotica* [40]. For the leg preparations without axonal tracing, 15 legs were isolated from first juvenile instars (5 forelegs, 5 midlegs, and 5 hindlegs) and fixed in 70% ethanol, dehydrated in ethanol (90%, 96%, 100%), and cleared in methyl salicylate. In total, 50 leg preparations from first juvenile instars were analysed (12 forelegs, 26 midlegs, and 12 hindlegs). 

The experiments documented in the present study comply with the principles of animal care of the Justus Liebig University Gießen, Germany, and also with the current law of the Federal Republic of Germany. 

### 2.2. Neuroanatomical Staining by Axonal Tracing 

The neuroanatomy of the subgenual organ complex in adult stick insects and first juvenile instars was stained by axonal tracing with cobalt solution [44]. The tracing was carried out on the nervus cruris, the main leg nerve from the thoracic ganglia [45,46,47]. The legs were cut off from the body and placed in a glass dish covered with Sylgard (Sylgard 184, Suter Kunststoffe AG, Fraubrunn, Switzerland). For adult insects, they were placed with the ventral side up, mounted with insect pins, and the glass dish was filled with *Carausius* saline to keep the tissue moist (177.96 mmol NaC1, 17.4 mmol KC1, and 25.1 mmol MgC1_2_ × 6 H_2_O, from Roth, Karlsruhe, Germany; 7.48 mmol CaC1_2_ × 2 H_2_O, from Merck, Darmstadt, Germany; and 1.98 mmol Tris, from Sigma-Aldrich, St. Louis, MO, United States; in Aqua dest., pH = 7.4) [48,49]. For the first juvenile instars, the legs were also pinned for the preparation, in this case with the lateral side facing upwards. For the axonal staining, the nervus cruris was located within the femur: the cuticle of the femur was cut open on the ventral side using a piece of a thin blade (Feather FA-10, Feather, Osaka, Japan, 0.1 mm), and the tendons and muscle tissue were carefully removed at the proximal femur. The nervus cruris was cut close to the distal end of the femur with iridectomy scissors. For the axonal tracing, the cut end of the nerve was placed in a glass capillary filled with 5% cobalt solution (CoCl_2_ × 6 H_2_O; Merck, Darmstadt, Germany) dissolved in Aqua dest. For the tracing, the legs were then incubated in a moist chamber at 4 °C for 24 h. The intracellular staining was processed with a solution of ammonium sulphide (Alpha Aesar, Karlsruhe, Germany; 1% in *Carausius* saline), which stains the neuronal structures that have taken up the cobalt solution. Leg preparations were incubated for 12 min, rinsed in *Carausius* saline, and fixed in chilled paraformaldehyde (4%, from Sigma Aldrich, St. Louis, Missouri, United States, in phosphate buffer, 0.04 mol/L Na_2_HPO_4_, and 0.00574 mol/L NaH_2_PO_4_ × 2 H_2_O; pH = 7.4) for 60 min at room temperature. Following fixation, the legs were dehydrated in a graded ethanol series (Carl Roth, Karlsruhe, Germany) and cleared in methyl salicylate (Merck, Darmstadt, Germany). 

### 2.3. Microscopy 

Leg surface: Midlegs from adult and first juvenile insects were cut off with scissors at the coxa. The legs were photographed under a Leica 9Si dissection microscope with an integrated camera (1024 × 768 pixel), and photographs were acquired in the Leica Application Suite version 4.12 (Leica Microsystems CMS GmbH, Wetzlar, Germany). 

Neuroanatomy of the subgenual organ complex: Leg preparations were viewed under an Olympus BH-2 microscope (Olympus, Shinjuku, Japan). They were mounted on a microscopy slide with the anterior side up and covered with methyl salicylate. Photographs were acquired with a Leica DFC7000 T camera (1920 × 1440 pixel) in the Leica Application Suite version 4.9 (Leica Microsystems CMS GmbH, Wetzlar, Germany). 

Preparations were photographed in series and usually assembled into stacked images by CombineZP. Figure panels were assembled and labelled in CorelDraw 11 (Corel, Ottawa, ON, Canada). 

### 2.4. Terminology of Leg Axis 

The terminology used for the axis of the tibia and femur and for the levels of sections of the tibia is according to Ball and Field (1981) [50]. 

### 2.5. Statistical Analysis

Statistical analysis was carried out in GraphPad Prism 4 (GraphPad, San Diego, CA, USA). The proportions of leg preparations were compared in a Chi-square test or a two-sided Fisher’s Exact test. 

## 3. Results

The midleg length in newly hatched *S. chlorotica* (Figure 2a) and in adult individuals differed by a factor of ca. 4×: the average length of the midleg tibia in first juvenile instars was 3.9 mm ± 0.2 mm (*n* = 10), whereas in adult females, the average length of the midleg tibia was 15.9 mm ± 0.8 mm (*n* = 16). The difference in size is shown in Figure 2b. 

For the subgenual organ complex, the membrane between the SGO and the DO was seen in adult *S. chlorotica* using light microscopy (*n* = 9; Figure 1d). It was best detectable in the lateral view. 

**Figure 3:**
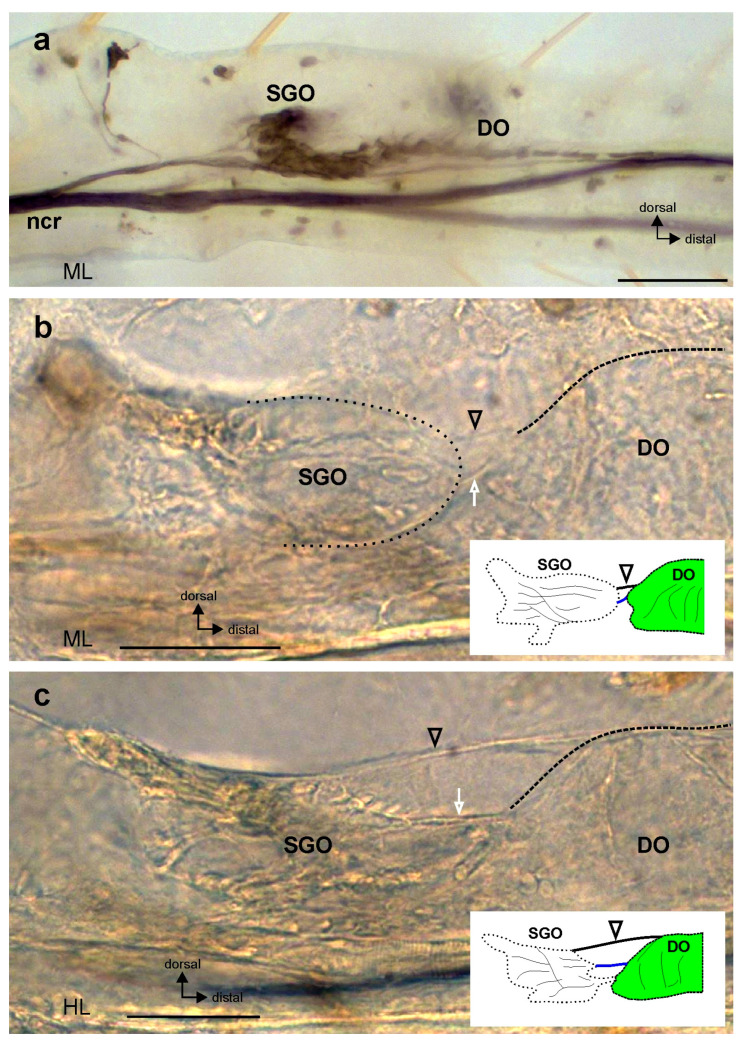
The connection between sensory organs in the subgenual organ complex. (**a**) The subgenual organ complex in *Sipyloidea chlorotica*, first juvenile instar, in the midleg. The subgenual organ complex consists of the subgenual organ (SGO) and distal organ (DO), stained by axonal tracing. (**b**,**c**) Connection between the SGO and DO by the membrane (white open arrow) and a dorsal strand of connective tissue (black open arrowhead). Insets show schematics of the preparations with colour coding following Figure 1b. The membrane between the SGO and DO is shown in blue, and the arrowhead indicates the dorsal strand of connective tissue; DO. Preparations show (**b**) a midleg and (**c**) a hindleg. All preparations are viewed from the anterior. Scale bars: (**a**) = 100 µm; (**b**,**c**) = 50 µm. Abbreviations: DO, distal organ; HL, hindleg; ML, midleg; ncr, nervus cruris; SGO, subgenual organ.

Through analysis of the neuroanatomy in the first juvenile instars, the subgenual organ complex (Figure 3a) was shown in all leg pairs of the newly hatched first juvenile instars. The membrane connection was also developed between the SGO and the DO (Figure 3b,c). As in the adults, the membrane was clearly seen in the lateral view (Figure 3b,c). The SGO extended distally in the direction of the DO (Figure 3b,c). In addition, a dorsal strand of connective tissue spans between the sensory organs (Figure 3b,c; black open arrowhead). 

**Figure 4:**
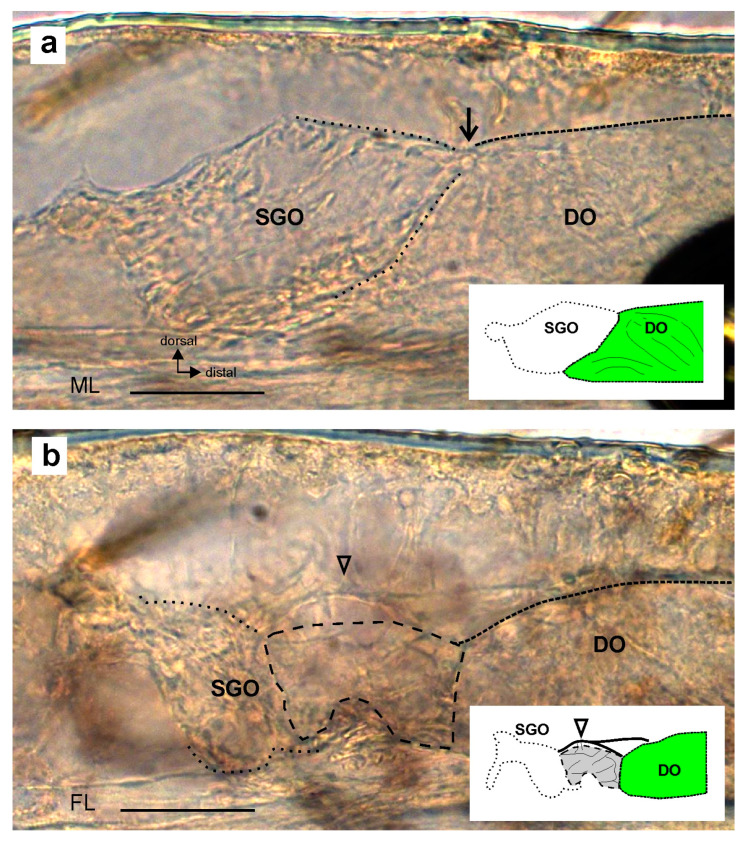
Preparations of the subgenual organ complex lacking the connecting membrane between the sensory organs. The membrane can be absent between the subgenual organ (SGO) and distal organ (DO) in cases where (**a**) the SGO and DO are in direct contact (indicated by black arrow) or (**b**) tissue is present between the sensory organs (outlined by hatched line). The dorsal strand of connective tissue is indicated by an open arrowhead in (**b**). Insets show schematics of the preparations with colour coding following Figure 1b. The grey mass in (**b**) shows the diffuse tissue between the SGO and DO. The preparations are viewed from the anterior. Scale bars = 50 µm. Abbreviations: DO, distal organ; FL, foreleg; ML, midleg; SGO, subgenual organ.

The membrane connection between the sensory organs, however, was not present in all preparations. It was absent in five leg preparations out of fifty, although the two sensory organs were formed in all of these cases. The morphology of the subgenual organ complex without the membrane was found in two principal forms. First, the connection was absent and there was a direct contact between the SGO and the DO (*n* = 3) (Figure 4a). Second, the space between the SGO and the DO was filled with tissue that was less compact than the SGO tissue (Figure 4b). In this case, the SGO tissue did not show the characteristic extension in the distal direction (*n* = 2). The preparations in which the connection was absent were obtained from all thoracic legs (foreleg: *n* = 1; midleg: *n* = 3; hindleg: *n* = 1). For all legs, the differences in the proportions of preparations without the membrane were not statistically significant (Chi-square test: *p* > 0.5, Chi-square = 0.7326, df = 2).

The proportion of legs with the membrane present was similar in the preparations from both axonal tracing experiments and direct ethanol fixation without intracellular staining (axonal tracing: 31/35 legs present; ethanol fixation: 14/15 legs present). The differences between these samples were not statistically significant (two-sided Fisher’s Exact test, *p* > 0.01), showing that the processing of the leg preparations had no influence on the detection of the membrane by light microscopy (e.g., by counterstaining of tissue during the axonal tracing procedure, as seen in the preparation in Figure 1d).

## 4. Discussion

### 4.1. The Morphological Connection between Sensory Organs and the Postembryonic Development of the Subgenual Organ Complex in Stick Insects 

This study documents the morphology and a connecting membrane between two chordotonal organs in first juvenile instars, to extend the knowledge on sensory neurons that are differentiated by the time of hatching [40]. It was hypothesized that the connecting membrane is also differentiated in newly hatched stick insects, as it contributes to the morphology of the sensory organ complex [29] and thus presumably to the sensory function. The results presented here show that the overall morphological organisation of the subgenual organ complex in newly hatched juvenile instars resembles the morphology in the adult stick insects (Figure 1d). The SGO, the DO, and the membrane between the two chordotonal organs are present already in newly hatched *S. chlorotica*. This includes the shape of the distal SGO, which extends towards the DO (Figure 1b,d and Figure 3). Presumably, the tissues associated with the scolopidial sensilla also differentiate during embryogenesis like the sensory neurons. This embryonic origin has been shown for the main attachment of the SGO to the inner leg cuticle [36]. The morphological findings indicate that the sensory organs might also respond physiologically to mechanical stimuli in early postembryonic stages, as they do in adults. The detection of mechanical stimuli in the habitat like substrate vibrations is important in postembryonic development, as in the adult stage. For stick insects, however, the role of substrate vibrations in their behaviours is not well understood so far, but it could include the detection of conspecifics as well as predators. 

The morphology of the sensory complex in general and the membrane in particular is not specific to *S. chlorotica* but is also documented for two other species of Old World stick insects (Oriophasmata [21]). A more detailed description of the membrane is given for two species of New World stick insects (Occidophasmata), *Oreophoetes peruana* (Saussure 1868) (Diapheromerinae) and *Peruphasma schultei* (Conle & Hennemann 2005) (Pseudophasmatinae) [29,30]. Therefore, this morphological feature is presumably common among species of the Neophasmatodea (combining Oriophasmata and Occidophasmata).

### 4.2. Functional Implications of a Connection between Chordotonal Organs in Juvenile Instars

For a mechanoreceptor organ, the coupling to other anatomical elements is crucial since these can provide the mechanical energy from activating inputs such as sound, vibration, or stretch [18,51]. In this respect, the functional morphology of chordotonal organs provides the basis for the biomechanics of sensory organs [27,52]. Hence, the connection between two chordotonal organs suggests that the sensory organs may be functionally coupled and therefore physiologically similar [29]. The position of both organs in the hemolymph channel allows for the detection of substrate vibrations transferred along the leg in the hemolymph. In bees, it was shown that the SGO moves within the tibia upon vibrational stimulation, responding also with changes in the SGO morphology [52,53]. 

In stick insects, the connection between the SGO and the DO seems to influence the shape of the distal SGO and very probably also its mechanics [29]. This is particularly noteworthy since the DO does not have strong attachments to the leg cuticle, as seen for the SGO in stick insects or bees [21,52]. Since the membrane connects to the DO surface in the longitudinal plane, this organisation may limit both the SGO displacement and also the lateral DO movement and could thus result in similar displacements of the sensory organs. This would provide a further argument for a vibrosensory role for the DO. An open question also concerns the physiological advantage of such a coupling since the SGO in a narrow hemolymph channel should be well stimulated through substrate vibrations [26,54,55]. The sensory physiology of the subgenual organ complex may be difficult to investigate by electrophysiology in first juvenile instars due to their small size, but it could be further analysed in adult *S. chlorotica*, where vibrational thresholds were recorded [42] (see below). 

### 4.3. Absence of the Membrane between Sensory Organs 

In a few leg preparations, the membrane between the sensory organs was absent. Two main morphological organisations were described (Figure 4). The absence of the membrane was also found in a few preparations in adults of other stick insects, *O. peruana* [29] and *P. schultei* [30]. The comparison between the three species shows that the proportion of preparations where the membrane is absent is slightly higher in *S. chlorotica* instars and in *P. schultei* than in *O. peruana* (*S. chlorotica*, first juvenile instars: 90%; *P. schultei*, adult insects: 89%, [30]; *O. peruana*, adult insects: 96% [29]). These differences in proportions between species are not statistically significant (Chi-square test: *p* > 0.1, Chi-square = 3.753, df = 2). It is likely that the membrane in these cases is not regularly differentiated during embryonic development [29]. The developmental mechanisms underlying the morphogenesis of the sensory organs, their attachment structures, or the membrane have so far not been analysed further. For example, it is not understood whether or not the sensory neurons in the SGO and in the DO differentiate from the same precursor cells [1,56] or originate separately, which could relate to their embryonic orientation and connection. 

The physiological influence of the presence or absence of the membrane could be analysed by recording vibrational thresholds of the subgenual organ complex in adult insects [30]. The morphology could be analysed histologically after such threshold recordings. In such structural analysis, not only the presence of the membrane but also the integrity of the SGO as a highly sensitive vibration receptor [26,57] should be documented. Most important would be the biomechanical properties of the membrane connection and a characterisation of the connection it provides between the two organs. It has been suggested that the link to the DO may result in more limited movements of the SGO along the main axis of the tibia [29]. Further physiological experiments may thus underline the unique organisation of the subgenual organ complex seen in stick insects. 

## Data Availability

The original contributions presented in this study are included in the article, further inquiries can be directed to the corresponding author.
